# Development and Implementation of Whole Genome Sequencing-Based Typing Schemes for *Clostridioides difficile*

**DOI:** 10.3389/fpubh.2019.00309

**Published:** 2019-10-24

**Authors:** Sandra Janezic, Maja Rupnik

**Affiliations:** ^1^National Laboratory for Health, Environment and Food, Maribor, Slovenia; ^2^Medical Faculty, University of Maribor, Maribor, Slovenia

**Keywords:** *Clostridioides (Clostridium) difficile*, wgMLST, cgMLST, typing, CDI, SNV

## Abstract

*Clostridioides difficile* is an important nosocomial pathogen increasingly observed in the community and in different non-human reservoirs. The epidemiology and transmissibility of *C. difficile* has been studied using a variety of typing methods, including more recently developed whole-genome sequence (WGS) analysis that is becoming used routinely for bacterial typing worldwide. Here we review the schemes for WGS-based typing methods available for *C. difficile* and their applications in the field of human *C. difficile* infection (CDI). The two main approaches to discover genomic variations are single nucleotide variant (SNV) analysis and methods based on gene-by-gene comparisons (frequently called core genome or whole genome MLST, cgMLST, or wgMLST). SNV analysis currently provides the ultimate resolution, however, typing nomenclature and standardized methodology are missing. On the other hand, gene-by-gene approaches allow portability and standardized nomenclature, and are therefore becoming increasingly popular in bacterial epidemiology and outbreak investigation. Two commercial software packages (BioNumerics and Ridom SeqSphere+) and an open source database (EnteroBase) for allele and sequence type determination for *C. difficile* are currently available. Proof-of-concept WGS studies have already enabled advances in the investigation of the population structure of *C. difficile* species, microevolution within the epidemic strains, intercontinental transmission over time and in tracking of transmission events. WGS of clinical *C. difficile* isolates demonstrated a considerable genetic diversity suggesting diverse reservoirs for CDI. WGS was also shown to aid in resolving relapses and reinfections in recurrent CDI and has potential for use as a tool for assessing hospital infection prevention and control performance.

## Introduction

*Clostridioides (Clostridium) difficile* is currently one of the most important human pathogens (1). The majority of *C. difficile* infections (CDI) is still identified or associated with the healthcare environment, though the incidence of community CDI is rapidly increasing. Because of its importance as a nosocomial pathogen, the development of different typing methods was needed to identify and control hospital transmissions and outbreaks. Several typing schemes were introduced for *C. difficile*; among early phenotypic methods serotyping was used widely, but subsequently replaced by pulsed-field gel electrophoresis (PFGE) and finally by PCR ribotyping which is the current gold standard for *C. difficile* typing (1–3). However, apart from multi locus sequence typing (MLST), standardization of all established typing methods has been difficult and inter-laboratory comparisons hampered (2).

Although these methods have contributed greatly to understanding of the epidemiology of CDI, they usually do not have sufficient discriminatory power to distinguish between closely related stains needed for outbreak investigations and to understand transmission events. With development of new sequencing methodologies, there is now the possibility to sequence and compare whole bacterial genomes and not rely only on a single or a few genomic loci to address the genetic relatedness of strains. Therefore, the genome-wide sequence analysis is now frequently used for molecular typing to provide accurate and reproducible investigation of the relatedness of isolates with the highest level of genetic resolution (4).

Here we will review studies on the development and implementation of typing methods based on whole genome sequencing (WGS) and their applications, focusing mainly on healthcare-associated CDI. Proof-of-concept studies have already demonstrated the general applicability of WGS-based typing for investigation of global and national surveillance of *C. difficile* epidemiology, and have expanded our understanding of transmission dynamics and recurrent infections. All these aspects will be reviewed here. However, use of WGS for strain characterization such as analysis of virulence and resistance gene pool and evolutionary aspects will not be covered in this review. *C. difficile* is commonly isolated also from animals and the environment and the paper by Knight and Riley in this special issue (5) will cover applications of comparative genomics from this perspective.

## Comparative Genomics and Two Different Approaches for WGS Typing

For the principles of next-generation sequencing technologies and bioinformatic processes, from the raw sequence data to the genomes, the reader is referred to other recent reviews (4, 6).

**Figure 1:**
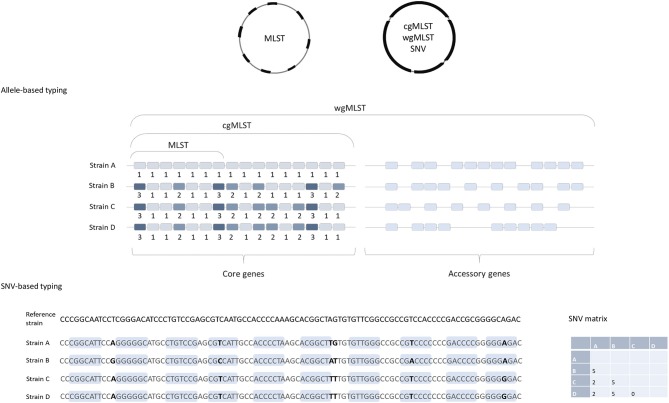
Comparison of allele-based and SNV-based typing approaches. cgMLST, wgMLST, and SNV approaches are based on the genome-wide analysis and MLST includes only seven housekeeping genes. Note that strains B, C, and D are identical in MLST approach (same allelic profile in seven genes) and both are the same MLST-ST; but they would differ in cgMLST, strain C and D having identical cgMLST allelic profile, and strain B differs from C and D in three additional genes. In the SNP-based approach, short reads are aligned to a reference genome and the nucleotide differences in both coding (light blue boxes) and non-coding regions (excluding horizontally acquired elements and putative recombination regions) are determined. The number of SNV differences between the pairs of isolates is presented in the matrix on the right.

To determine the genomic similarities and differences between investigated isolates (e.g., to determine which strains could be clonal) different comparative genomics approaches are available. They differ mainly in methodologies used, easiness of data sharing and their discriminatory power. Below we will briefly describe the two of most commonly used approaches for typing of isolates for epidemiological surveillance purposes. The first one is based on comparison of differences in single nucleotide polymorphisms (SNPs), also called single nucleotide variant (SNV) sites. The second approach is based on analysis of multiple genes across the whole genome, so called gene-by gene or allele-based approaches. This is also designated core genome (cg) or whole genome (wg) multi locus sequence typing (cgMLST or wgMLST) (Figure 1).

### SNV Approach—When Are Two Strains Clonal?

Strain typing based on core genome SNVs (cgSNVs) is currently considered as a method with very high discriminatory power, since it allows us to distinguish between isolates if their genomes differ in a single nucleotide (7). In this approach, short reads (data generated from sequencing of short genomic fragments) or assembled contigs (longer contiguous sequences of overlapping reads) are mapped against the genome of a reference strain to identify differences in coding and non-coding regions. This process is named variant calling (8). The pipeline that has been widely used for SNV analysis of *C. difficile* includes mapping of short reads to a reference genome, variant calling, filtering of high quality SNVs, and identification and removal of putative recombination regions. The result is a concatenated set of high quality SNVs present in the core genome (part of genome that is common to all comparing isolates). The number of SNVs is subsequently used to asses genetic relatedness of isolates (9–11). Relationships between isolates can be visualized by constructing phylogenetic trees to help us understand transmission networks.

The choice of the reference strain can have significant impact, especially on the resolution of SNV-based approaches. The reference strain should be closely related to the isolates included in the comparison since only the regions present in the reference strain will be used for variant calling. Therefore, the more distant the reference sequence the more regions will be omitted from the analysis. Also, a standardized nomenclature would be difficult to adopt since there are multiple algorithms used to analyze SNVs. For this reason, SNV calling is a very useful method for local transmission analysis but not as appropriate for global strain comparisons, unless the genome sequences are made publicly available. However, in this case, the genomes still need to be (re)analyzed locally (8).

The commonly adopted way to determine relatedness of strains in the SNV approach is to count the number of SNV differences between two sequences (SNV threshold). However, it is important to note that proposed criteria of SNV relatedness should not be taken as the absolute rules but should be considered as a guide (8). To determine the SNV threshold, it is important to know the evolutionary rates, i.e., the rate at which the particular bacterial genome evolves (12). This can be estimated from longitudinal sampling from infected individuals and then assessing the number of accumulated substitutions in the genome over time (9).

By comparing genome sequences of the first and the last isolate obtained from individual patient (samples were collected at a median interval of 51 days), an evolutionary rate of 0.74 SNVs (95% confidence interval, 0.22–1.40) per genome per year and a mean within-host diversity of 0.30 SNVs (95% CI, 0.13–0.42 SNVs) were determined, in the study by Eyre et al. (10). Similar estimations of *C. difficile* evolutionary rates were obtained in other studies, either by using serial samples from the patients with recurrent or on-going CDI and/or in *in vitro* gut models (9, 10, 13). By using this prediction of evolutionary rate, the guideline for two isolates being clonal, or genetically related (are most probably a result of direct transmission), is that there are ≤2 SNVs between their sequenced genomes. For genetically unrelated isolates ≥10 SNVs are expected (10). This SNV relatedness criterion has now been widely accepted for transmission networks and outbreak investigations, and used in several studies that will be presented later in this review.

### Gene-by-Gene Comparison, cgMLST, and wgMLST

Cg- or wgMLST typing works on the same principles as the classical MLST, described by Maiden et al. (14), a comparison of strains based on sequence differences in a pre-defined set of housekeeping genes/loci. Usually seven housekeeping genes/loci are included in MLST schemes for most bacteria, including *C. difficile* (15). For each of the seven loci, the different sequences are assigned distinct allele numbers and the alleles at each genes are described as the allelic profile (Figure 1). Finally, for each allelic profile (the series of seven allele numbers) a unique sequence type (ST) is determined (14).

Because only a small number of genes are included in the analysis, MLST usually does not have sufficient discriminatory power to differentiate between closely related strains, e.g., strains that belong to the same PCR ribotypes, which makes this method insufficient for investigations of transmission events. To overcome this, an extension of MLST using a genome-wide gene-by-gene allele calling of hundreds or thousands of loci, so-called cgMLST and wgMLST was developed (16). The cgMLST scheme utilizes comparison of the non-repetitive genes that are conserved in all the members of a species, so called core genes. On the other hand, wgMLST examines a greater number of loci, and includes accessory genes as well as the core genes; these are genes that are variably present across a species (Figure 1), including also repetitive genes and pseudogenes (4).

### Available Schemes for *C. difficile* WGS-Based Typing

For *C. difficile*, three publicly available schemes are available for cg- and/or wgMLST typing, and analysis can be performed either by using commercial software (BioNumerics, Applied Maths or SeqSphere+, Ridom) or by a freely accessible online resource (EnteroBase). Additional new schemes are being developed (https://www.biorxiv.org/content/10.1101/686212v1?rss=1). The cgMLST scheme for *C. difficile* include 2270 loci (60.4% of the genes present in strain 630; SeqSphere; https://www.cgmlst.org/ncs/schema/3560802/) (17). wgMLST is available in Enterobase and in BioNumerics where, together with 1,999 core genes, another 6,713 accessory genes are included in the analysis http://www.applied-maths.com/sites/default/files/extra/Release-Note-Clostridium-difficile-schema.pdf.

The advantage of cgMLST and MLST is that sequences and allelic profiles of strains can be compared via the internet with central databases enabling uniform typing nomenclature that facilitate international comparability of typing data (16, 17). On the other hand, wgMLST might offer greater resolution between closely related strains, but the nomenclature is not standardized. However, EnteroBase contains all publically available genomic sequences (uploaded from public archives and assembled into annotated draft genomes), and therefore wgMLST data can be compared to all previously published *C. difficile* genomes and interpreted within a global context (18).

In contrast to SNV, the allele-based approaches do not need the genome of a closely related reference strain for the initial alignment of reads or contigs. Also, in the allele-based approach, both mutation (usually resulting in a single SNV) and recombination (that is more likely to introduce multiple deletions or insertions within allele) are counted as a single evolutionary event, meaning that there is no need to apply additional steps to identify and remove putative recombination regions (9, 19).

To test the discriminatory power and applicability of cgMLST to differentiate closely related strains, Bletz et al. (17) reanalyzed data from published outbreak investigations. With cgMLST they were able to differentiate among epidemiologically related strains and the conclusions were in concordance with the published SNV analysis. By re-analyzing two different outbreak investigations and considering the guide for number of SNV expected in genetically unrelated and related isolates (≥10 SNV and <2 SNVs, respectively) (10), the authors proposed a threshold of ≥7 alleles difference for strains being unrelated and ≤6 alleles for strains that are likely to be clonal. With this threshold, the cgMLST predicted the same clusters of related strains as SNV analysis. All strains within the defined threshold were assigned to the same cgMLST cluster type (CT) (17).

## Current Implementation of WGS Typing in Human CDI

The feasibility of using WGS of *C. difficile* genomes on benchtop sequencing platforms for transmission investigation to rapidly distinguish between outbreak and non-outbreak cases in a clinically relevant timescale was first demonstrated in 2012 in a pilot study conducted by Eyre et al. (20). Since then SNV-based analysis has been widely adopted for CDI surveillance and has revealed some novel understandings about transmission dynamics and recurrent infections (Table 1).

**Table 1:** WGS-based studies of *C. difficile* transmissions, outbreaks, or recurrences.

**References**	**Aim**	**Country**	**Description**
Didelot et al. (9)	Transmission	UK	Microevolutionary analysis of *C. difficile* (assessment of within-host evolutionary rate) and use of whole-genome sequencing for studying *C. difficile* transmission.
Eyre et al. (20)	Transmission	UK	A proof-of-principle study to investigate potentials of benchtop sequencers in routine clinical practice to investigate transmissions. Example of small cluster of genetically (MLST) identical *C. difficile* strains that could be differentiated with WGS.
Eyre et al. (10)	Transmission	UK	Investigating the role of symptomatic patients in the transmission of *C. difficile*. Study also demonstrates that in the settings with standard infection control most cases of infections are acquired from other sources, not symptomatic cases.
Eyre et al. (21)	Mixed infections	UK	Describing new algorithm for detection of mixed CDI in clinical samples from whole genome sequencing data.
Eyre et al. (22)	Transmission	UK	Investigating the role of asymptomatic patients in the transmission of *C. difficile*.
Eyre et al. (23)	Recurrence	UK	Use of WGS to determine if the reductions in recurrence of CDI observed with fidaxomicin occurred by preventing relapse, reinfection or both. Study demonstrated that fidaxomicin was superior to vancomycin in treating recurrent CDI.
Mac Aoga'in et al. (24)	Recurrence	Ireland	Use of WGS of *C. difficile* to discriminate between relapses and reinfections, and putative patient-patient transmission events in Ireland.
Kumar et al. (25)	Transmission	UK	A WGS to track the transmission of *C. difficile* PCR ribotype 027 within single hospital in UK, and to distinguish between the relapses and reinfections.
Sim et al. (26)	Recurrence	USA	Use of WGS to determine the rate of relapse and reinfection in patients with recurrent CDI.
Mawer et al. (27)	Transmission	UK	Exploring the role of symptomatic patients that are toxigenic strain positive but fecal toxin negative in transmissions of *C. difficile*.
Eyre et al. (28)	Transmission	UK	Use of WGS as surveillance tool to assess infection control effectiveness in hospitals by identifying the extent of hospital-acquired CDI transmissions within hospitals.
Stoesser et al. (29)	Transmission	UK	Investigation of genetic overlap of infant and regional *C. difficile* strains in Oxfordshire.
Donskey et al. (30)	Transmission	USA	Transmission of *C. difficile* from colonized or infected long-term care facility residents.
Endres et al. (31)	Outbreak	USA	Environmental transmission of *C. difficile* PCR ribotype 027 at a long-term care facility.
Eyre et al. (32)	Transmission	UK	WGS to analyze distinct patterns of *C. difficile* PCR ribotype spread across Europe.
Halstead et al. (33)	Transmission	UK	WGS to investigate if asymptomatic carriers contribute to nosocomial CDI.
Isidro et al. (34)	Outbreak	Portugal	Genomic investigation of *C. difficile* PCR ribotype 017 outbreak strains.
Kociolek et al. (35)	Transmission	USA	Transmission of CDI among symptomatic children.
Kong et al. (36)	Transmission	Canada	Investigation of transmission patterns between infected and colonized patients.
Williamson et al. (37)	Transmission	USA	Transmission of PCR ribotype 027 within healthcare facility and comparison to global collection of ribotype 027 isolates.
García-Fernández et al. (38)	Transmission	Spain	Routes and frequencies of transmission of *C. difficile* in a tertiary-care hospital in Madrid.

### Source Identification for Hospital CDI Cases

Traditionally, most cases of CDI have been thought to be acquired within the hospital environment, where transmissions occurs by horizontal spread from symptomatic patients (39, 40). However, assessment of CDI transmission in hospital settings by classical genotyping approaches was hampered by the low discriminatory power of used methods and by the number of patients that carry endemic genotypes, either PCR ribotypes or STs (9).

To assess the role of symptomatic patients in the transmission of *C. difficile* in the hospital environment Eyre et al. (10), sequenced genomes of *C. difficile* isolates from 1,223 patients with CDI. In this study, only 35% (*n* = 333) of isolates could be genetically linked (had ≤2 SNV) to at least one other isolate from a symptomatic patient and for 36% (*n* = 120) of these cases no plausible epidemiological link could be identified. Isolates from almost half (45%) the patients were genetically unrelated (≥10 SNPs) to any other previous case, meaning that these patients had likely acquired *C. difficile* from sources other than symptomatic patients. These findings suggest that there are rather diverse reservoirs of *C. difficile* and that transmissions other that those occurring between symptomatic patients within the hospital settings should be considered (e.g., asymptomatic patients, animals, households, and environmental sources) (10).

The role of asymptomatic patients in the transmission of *C. difficile* was explored by WGS in another study conducted by Eyre et al. (22), which demonstrated that although asymptomatic carriage is common, transmission from asymptomatic carriers is likely to be infrequent. In a similar Canadian study, slightly higher linkage rates were reported, where 46 and 52% of CDI cases could be linked to previous symptomatic and infected or colonized patients, respectively (36).

A study conducted in a single hospital demonstrated that a diverse set of isolates can be found also among children with CDI and that *C. difficile* transmissions, direct or indirect, between children with CDI are even less frequent (12.5%) than transmissions among adult CDI patients (35).

Several other studies have also addressed the questions of importance of other non-hospital reservoirs in *C. difficile* transmission and are reviewed in more details by Knight and Riley in this issue (5).

### Use of WGS for Study of CDI Recurrences

Within 2 months after treatment of an initial CDI episode, up to 25% of patients develop recurrent infection (41). Recurrent infection can be due to reinfection (CDI caused by newly acquired strain) or relapse (CDI caused by the original strain). Discrimination between relapses and reinfection usually does not have direct clinical implications and will not affect treatment. However, it might be important for controlling CDI, either through interventions to manage *C. difficile* transmission, or treatment policies (25). Several studies have already demonstrated usefulness of WGS comparisons in understanding the epidemiology of CDI recurrences (23–26). In these studies, the authors used similar approaches as described for transmission studies. In case of reinfections, isolates from the initial and following episodes were expected to be genetically unrelated, differing ≥10 SNVs, and in case of relapses, the isolates would be clonal, differing in ≤2 SNVs (23). All studies that explored the source of recurrent infection demonstrated that the majority of recurrent episodes are caused by primarily infecting strain, meaning that relapses are more common than reinfections (23–26).

## Backward Compatibility Between WGS and MLST

Currently, an assortment of classical and WGS-based typing methods is used for investigations of *C. difficile* epidemiology (2). Reverse compatibility of WGS with traditional typing methods is therefore important to compare the genotypes obtained with different approaches and to compare newly sequenced strains to existing and historical strains (42). From WGS data, seven MLST loci can be easily extracted to determine the allelic profile and ST. For ST calling directly from draft genomes a publically available PubMLST.org database can be used (43). SeqSphere and BioNumerics also enable ST determination directly from WGS data.

## Why Can PCR Ribotype not be Determined With WGS

PCR ribotyping has become a method of choice for typing of *C. difficile* in the majority of laboratories (2, 44). The method is based on analysis of banding patterns of PCR-amplified internal transcribed spacers (ITS) located between 16S and 23S rRNA genes in ribosomal operon. In *C. difficile*, as in many other bacteria, the ribosomal operon is present in several copies in the genome and different copies differ in the length of ITS (45) and, due to intraspecific diversity of ITS, PCR ribotyping is a good method for *C. difficile* genotyping (2).

In contrast to MLST-ST, PCR ribotype cannot be directly determined from WGS. Regions that are amplified in PCR ribotyping are repetitive and it is not possible to map short sequence reads generated by NGS correctly to such repetitive and modular regions (45, 46). To assign a PCR ribotype to a new ST or cgMLST cluster type, a representative strain would still need to be PCR ribotyped. But with the advances in NGS technologies (e.g., PacBio and Nanopore), read lengths are continually increasing (4). The availability of very long and very precise sequences will ultimately enable the *in silico* PCR ribotyping.

The ability to predict PCR ribotypes from whole genome sequencing data remains controversial. While the genome sequences of strains belonging to the same PCR ribotype mostly group together, it is important to appreciate the differences between a true ‘PCR ribotype determination' and ribotype inferred from genome sequencing data. Firstly, while grouping of strains with identical PCR ribotype is to be expected, there are exceptions and similarity of genome sequences of two different PCR ribotypes has been documented (36 and unpublished data). Secondly, due to limitations of short read sequencing explained above, comparison of two genomes shows only similarities in large part of genome, but not necessarily in the regions that are actually used for PCR ribotyping (i.e. ITS). Therefore, it is important to differentiate between ribotypes determined by actual PCR ribotyping and putative PCR ribotypes based on genome similarity, but excluding rDNA regions.

## Conclusion

WGS-based typing methods offer an excellent platform with high resolution and reproducibility that enable studies of both transmission and epidemiology of CDI, as well as positioning strains within the global population. However, especially for the understanding of global CDI epidemiology, whole genome data availability, either by sharing raw data or allelic profiles through freely accessible databases that support direct comparison of isolates is of paramount importance.

## Author Contributions

SJ and MR both contributed to the conception and writing of the paper.

### Conflict of Interest

The authors declare that the research was conducted in the absence of any commercial or financial relationships that could be construed as a potential conflict of interest.
